# Epidemiological profile, clinical characteristics, and surgical outcomes of genital fistula repair in Kinshasa, Democratic Republic of Congo: FisPro DRC Experience

**DOI:** 10.11604/pamj.2026.53.178.50433

**Published:** 2026-04-30

**Authors:** Lucien Wasingya Kasereka, Patricia Mavuzi, Tresor Kibendelwa, Gabriel Mathe Muhini, Augustin Kanyali, Jean-Paul Kituli Muhawali, Dieumerci Kaseso, Albin Baraka Munyanderu

**Affiliations:** 1 Department of Gynecology and Obstetrics, Medical Center Bolamu, Kinshasa, Democratic Republic of Congo,; 2 Institut Supérieur des Techniques Médicales, Kinshasa, République Démocratique du Congo,; 3 Fistula Program DRC, Kinshasa, Democratic Republic of Congo,; 4 Département de Chirurgie, Centre Hospitalier Mère et Enfant Monkole, Kinshasa, République Démocratique du Congo,; 5 Department of Surgery, General Referral Hospital of N´djili, Kinshasa, Democratic Republic of Congo,; 6 Department of Gynecology and Obstetrics, Butembo Fistula Hospital, Butembo, Democratic Republic of Congo,; 7 Department of Surgery, Catholic University of Graben, Butembo, Democratic Republic of Congo,; 8 Department of Public Health, Official University of Rwenzori, Butembo, Democratic Republic of Congo

**Keywords:** Epidemiology, clinical profile, genital fistulas, Kinshasa, Democratic Republic of Congo

## Abstract

The objective of this study was to present the epidemiological profile, clinical characteristics, and surgical outcomes of women operated on for genital fistulas at the Medical Center Bolamu in Kinshasa by the Fistula Program DRC (FisPro DRC) from 2021 to 2025. It was a retrospective, descriptive analysis that included 336 women who underwent genital fistula repair between January 1, 2021, and June 30, 2025. Data were collected from patients´ medical records. The variables included demographics, fistula etiology, anatomoclinical diagnosis, and post-operative status assessed at the 14-day postoperative period before discharge. Statistical analysis was performed using SPSS version 18.0 (SPSS Inc., Chicago, IL, USA). In our study population of 336 patients, the mean age was 42 years, and the average duration of the fistula was 9 years. A vast majority of patients (92.6%, n=311) had no prior surgical repair attempt. The primary etiology was obstetrical (67.0%, n=225), followed by iatrogenic causes (32.7%, n=110). Anatomically, the most prevalent types were vesicovaginal fistula (VVF) (40.8%, n=137) and perineal tears (40.5%, n=136). Following repair, a successful surgical outcome (closed and continent) was observed in 92.9% of cases (n=) at the 14-day postoperative assessment. Genital fistula is a real health challenge in Kinshasa, characterized by late presentation and severe lack of treatment access. The fistulas are caused particularly by obstetric and iatrogenic trauma. FisPro's surgical and functional success shows why we need dedicated fistula repair programs and stronger awareness and prevention.

## Introduction

According to the International Federation of Gynecology and Obstetrics (FIGO), a genital fistula is an abnormal connection between the genital tract and the urinary or gastrointestinal tract, leading to the leakage of urine or feces [1]. The most reported causes are obstetric complications from obstructed labor. Other causes are iatrogenic, congenital, and sometimes trauma from rape [2]. The resulting uncontrolled, constant leakage of urine and/or feces inflicts severe physical, psychological, and social consequences, leading to profound stigma, isolation, and marginalization for the affected women [3]. It remains a considerable health challenge in low-income countries in sub-Saharan Africa (SSA) and South Asia; in particular. The Democratic Republic of Congo (DRC) is among the countries bearing the world's highest burden of genital fistulas [2], a problem severely compounded by political instability, conflicts, and systemic underdevelopment of health infrastructure. In 2007, it was estimated that the country had approximately 14,200 women with existing symptoms of fistula [4]. Recent data in 2025 by the United Nations Population Fund (UNFPA) indicate that the number of women with fistula is more than 42,000 cases that are not treated [5]. Eastern DRC is a region where genital fistulas are more commonly reported, due to different challenges related to insecurity and poor health infrastructure. Other areas seem not to be concerned, yet the problem is present in the whole country, especially if obstetric complications are considered [6]. According to the Fistula Care Plus Project in 2019, genital fistulas are an underreported health challenge in Kinshasa and neighboring provinces. Factors like young age (20-29), being primiparous, and having limited education are associated with a higher risk [7]. In fact, the region of Kinshasa still lacks important information on epidemiology, clinical presentation and surgical outcomes of the patients with genital fistula, compared to other studies conducted in the Eastern part of the country [2,8,9]. The objective of this study was to describe the epidemiological profile, the clinical characteristics, the anatomical types, and the outcome of surgical patients operated on for genital fistula at Medical Center of Bolamu, in Kinshasa, from 2021 to 2025.

## Methods

This study was a retrospective, descriptive analysis, which included 336 women who underwent surgical repair for genital fistula at the Medical Center Bolamu in collaboration with FisPro DRC, in Kinshasa, from January 1, 2021, to June 30, 2025. The patients´ recruitments were carried out following four surgical campaigns from January 1, 2021, to July 2023, and full-time routine from July 2023 to June 2025. Medical Center Bolamu is a permanent fistula repair specialized center located in Kinshasa, Democratic Republic of Congo, which collaborates with Fistula Program DRC (FisPro DRC), a fistula repair non-profit non-governmental organization (NGO). It treats women with genital fistulas free of charge, via surgical outreaches then routinely, in Kinshasa and in Butembo, North-Kivu since 2021. This research included only the cohort of patients who were operated on in Kinshasa at the Medical Center Bolamu from January 1, 2021, to June 30, 2025. The sample was exhaustive. The data were collected from the patients´ records and the variables included the demographics, history, clinical diagnosis, surgical procedure protocol, and outcome at discharge. This concerns the first consultation and the first surgery by FisPro. The final study population was 336 cases. The data were primarily encoded in Microsoft Excel 2021, and the statistical analysis was performed using SPSS version 18.0 (SPSS Inc., Chicago, IL, USA). The study was conducted after the approval of the Ethics Committee, and all the patients signed informed consent about the procedure and the fact that their data would be included in the research process.

## Results

Our study included 336 patients. The cohort was particularly adult, with the largest proportion of patients being over 40 years old (49.1%, n=165). The majority of cases were chronic, 1 to 5 years (40.8%, n=137) or over 5 years (36.0%, n=121). Most of the patients (92.6%, n=311) had benefited from no previous fistula repair attempt. The experienced types of incontinence were nearly evenly split between urinary (50.9%, n= 171) and fecal (47.3%, n=159), with a smaller number experiencing both (1.8%, n=6). Crucially, the predominant etiology of the fistulas was obstetrical (67.0%, n=225), followed by iatrogenic causes (32.7%, n=110) (Table 1). The most prevalent fistula types were Vesicovaginal Fistula (VVF) (40.8%, n=137), and the perineal tear (40.5%, n=136), together constituting 81% of all cases. The others were recto-vaginal fistula (RVF) (7.7%, n=26), urethro-vaginal fistula (4.8%, n=16), ureteric fistula (2.7%, n=9), VVF associated with bladder stone (0.6%, n=2), VVF and vesico-cutaneous fistula (0.6%, n=2), total urethral loss (0.3%, n=1) and incontinence after repair (1.8%, n=6) (Table 2). There was a high surgical success rate, with 312 patients (92.9%, n=312) closed and continent, and 24 patients (7.1%, n=24) closed and incontinent (Table 3). While anatomical closure was achieved for all patients at discharge, residual urinary incontinence persisted in a small subset of the total cohort.

**Table 1:** sociodemographic profile and clinical characteristics of patients with fistulas

Variables	Characteristics	N	%
Age of the patients	<20 years	13	3.9%
	20-40 years	158	47.0%
	>40 years	165	49.1%
	Total	336	100.0%
Age of the fistula	< 1 year	78	23.2%
	1-5 years	137	40.8%
	>5 years	121	36.0%
	Total	336	100.0%
Previous surgical repairs outside FisPro	0	311	92.6%
	1	12	3.6%
	>1	13	3.9%
	Total	336	100.0%
Types of incontinence	Fecal	159	47.3%
	Urinary	171	50.9%
	Both	6	1.8%
	Total	336	100.0%
Etiologies of the fistula	Obstetrical	225	67.0%
	Iatrogenic	110	32.7%
	Congenital	1	0.3%
	Total	336	100.0%

**Table 2:** anatomoclinical diagnosis and classification of fistula types

Variable	Characteristics	N	%
Type of the fistula	FVV*	137	40.8%
	RVF**	26	7.7%
	VVF et RVF	1	0.3%
	VVF and vesico-cutaneous fistula	2	0.6%
	Perineal tear	136	40.5%
	Incontinence after repair	6	1.8%
	Ureteric fistula	9	2.7%
	VVF and bladder stone	2	0.6%
	Uretro-vaginal fistula	16	4.8%
	Total uretral loss	1	0.3%
	Total	336	100.0%

* VVF: vesicovaginal fistula; **RVF: rectovaginal fistula

**Table 3:** surgical outcomes of patients after genital fistula repair, evaluated at the 14-day postoperative discharge assessment

Variable	Characteristics	N	%
Outcome	Closed, continent	312	92.9%
	Closed, incontinent	24	7.1%
	Total	336	100.0%

In our study, the mean age at presentation was 42 years (range 15 to 78 years), with 49.1% aged above 40 years. The average duration of the fistula before consultation was 9 years, with a standard deviation of 11 years. The study population presented almost the same fistula characteristic with other cohorts reported in the country, especially a reported fistula chronicity, with the mean duration of the fistula ranging from 5 to 10 years before surgical repair [8,10,11]. Nevertheless, the age of the patients was not homogenous, with a mean age of 27,96 years reported in Katanga [11], 33.24 years reported in North-Kivu [8] and 35.20 years in the northern part of the country. The age of patients with fistula, especially the onset, may be related to the diversity of reproductive maternal activities in different areas, with unmanaged obstetrical complications considered the main cause. Even though the dispersion is present in the mean age of patients in the country, one fact remains persistent and common: the chronic duration of the fistula before surgical repair. This may partially be explained by lack of awareness and scarcity of facilities where patients can benefit from fistula repair. Compared with other countries, by the way, there was a reported fistula duration before surgery a bit lower in Uganda 3 years [12] and in Ethiopia 5 years [13], but 10 years was reported in Northern Tanzania [14].

## Discussion

In our cohort (Table 1), the majority of the patients (92.6%, n= 311) had not benefited from a prior attempt at fistula repair. The large population and the rarity of specialized fistula care programs in Kinshasa may explain this. From 2017 to 2019, only the Saint Joseph Hospital could organize fistula repair campaigns for the whole population of Kinshasa, more than 17 million inhabitants [7]. This could not cover and reach all the cases, as a temporary periodic outreach. There is still a need to strengthen the fistula repair program in the region and scale up awareness and patients´ accessibility. Furthermore, fistula chronicity is a challenge for repair. Long-standing fistulas are invariably associated with severe tissue loss, dense scarring, and extensive fibrosis [14].

At the presentation, the types of incontinence experienced by the patients were fecal incontinence (47.3%, n=159) and urinary incontinence (50.9%, n=171) (Table 1). Regarding the different etiologies, obstetrical fistula was the most prevalent (67.0%, n=225) and iatrogenic fistula (32.7%, n=110). This was generally similar to other cohorts in sub-Saharan Africa [15] reporting a proportion of obstetric fistulas as the most prevalent etiology (60-90%) among women with genital fistula throughout the continent. This may be different in other continents. In Asia, for example, etiologies of genital fistulas reported in an Indian cohort were in the following proportions: obstructed labor (33.3%), lower-segment cesarean section (23.3%), hysterectomy (23.3%), and radiotherapy (10%) [16].

The prevalent anatomical types of fistulas in our population (Table 2) were the vesico-vaginal fistula (VVF) (40.8%, n=137) and perineal tear (40.5%, n=136). While VVF overwhelmingly dominates large African cohorts, often comprising 70-80% of genital fistulas and causing primary urinary leakage [13,17,18], the considerable proportion of perineal tears in our cohort suggests a high prevalence of low genital trauma resulting from poorly managed fetal deliveries. In low proportions, our patients presented other anatomical types of fistula: recto-vaginal fistula (RVF) (7.7%, n=26), urethro-vaginal fistula (4.8%, n=16), ureteric fistula (2.7%, n=9), VVF associated with bladder stone (0.6%, n=2), VVF and vesico-cutaneous fistula (0.6%, n=2), total urethral loss (0.3%, n=1) and incontinence after repair (1.8%, n=6). Managing these morbidities, rectal defects, severe perineal scarring, and urethral deficiency requires particularly sophisticated reconstructive techniques [19], requiring a specialized trained fistula surgeon.

At discharge of our patients, the 14-day post-operative closure rate was (92.9%, n=312), assessed as closed and continent (Table 3). Such success may be related to superior surgical technique, meticulous pre-operative assessment, comprehensive perioperative and post-operative management, and adherence to surgical principles. In fact, at Bolamu Medical Center, FisPro DRC counts well-trained personnel: one fistula surgeon and two trainees, and nurses, with full-time follow-up of patients. Nevertheless, the assessment was intermediate. There is still a need to assess this rate over a prospective long-term period.

## Conclusion

This study, encompassing 336 women who underwent genital fistula repair at the Medical Center Bolamu in Kinshasa between 2021 and 2025, confirms that genital fistula is a present, chronic, and underreported health challenge in Kinshasa, DRC, driven primarily by obstetric complications and complicated by long delays in accessing care. The mean duration of fistula before consultation was 9 years, and a significant majority (92.6%) had no prior surgical repair attempts, indicating severely limited awareness and scarcity of fistula repair care. Vesicovaginal Fistula (VVF) (40.8%) and a high prevalence of perineal tears (40.5%) were noted, pointing to issues with poorly managed deliveries across facilities. At the 14-day post-operative, the closure and incontinence rate was 92.0%. This success demonstrates the feasibility and impact of specialized fistula repair programs. Nevertheless, regarding the population and chronicity of the condition, there is still a need to scale up the fistula repair network and enhance public awareness. Besides, it is important to integrate quality obstetric care to prevent new cases.

### What is known about this topic

- Genital fistulas are due to obstetrical complications in poor health conditions, more in rural areas;
- Genital fistulas are more commonly reported in Eastern DRC, a war-affected zone;
- Vesicovaginal fistulas are the most prevalent reported diagnosis.

### What this study adds

- Genital fistulas are present in major urban cities and remain a chronic condition in the population;
- Perineal tears grade III and IV are as prevalent as vesicovaginal fistulas.
